# Supplementary material for: Household Air Pollution and Angina Pectoris in Low- and Middle-Income Countries: Cross-Sectional Evidence from the World Health Survey 2002–2003
Source: Int J Environ Res Public Health. 2020 Aug 11;17(16):5802. doi: 10.3390/ijerph17165802 (PMC7460098; doi:10.3390/ijerph17165802)
Supplement: Supplementary file 1 [file ijerph-17-05802-s001.pdf]

Supplementary tables

Adjusted [a] associations (Odds ratios and 95% confidence intervals) of angina pectoris [b] with type of cook and heating fuels used in household.

Table S1. Angina diagnosed.

Characteristic		Africa		Americas		Asia		Europe		Pooled sample	
		**OR**	**(95% CI)**	**OR**	**(95% CI)**	**OR**	**(95% CI)**	**OR**	**(95% CI)**	**OR**	**(95% CI)**
Household fuel used for cooking or heating 1	Gas	1		1		1		1		1	
	Electricity	0.99	0.67–1.45	0.09	0.02–0.32	0.10	0.52–1.91	0.28	0.20–0.41	0.54	0.42–0.69
	Kerosene	0.68	0.28–1.63	n/a	n/a	1.46	0.92–2.32	0.22	0.13–0.35	0.71	0.50–1.01
	Coal/charcoal	1.63	1.15–2.31	1.48	0.71–3.12	0.59	0.44–0.79	0.83	0.31–2.19	0.75	0.60–0.94
	Wood	2.24	1.68–2.99	0.83	0.58–1.20	1.00	0.77–1.30	0.89	0.48–1.64	1.11	0.92–1.33
	Agriculture/dung/ shrubs/other	0.74	0.36–1.56	0.91	0.22–3.83	0.93	0.60–1.44	0.45	0.09–2.16	1.01	0.68–1.50
	Mixed solid/liquid/gas/el ectricity	1.45	0.90–2.34	1.28	0.71–2.30	0.68	0.48–0.96	0.80	0.55–1.14	1.00	0.79–1.27
	Mixed solid only	1.80	1.15–2.81	5.75	1.62–20.47	0.10	0.41–2.42	1.77	0.27–11.83	1.21	0.76–1.93
	Mixed liquid/gas/electrici ty only	0.94	0.38–2.33	1.64	0.61–4.44	0.80	0.49–1.29	0.57	0.34–0.96	0.89	0.56–1.40
Household fuel used for cooking or heating 2	Gas/electricity/ker osene	1		1		1		1		1	
	Coal/charcoal/woo d/agriculture/crop /shrub/ grass	2.11	1.65–2.70	0.95	0.69–1.34	0.90	0.71–1.14	1.05	0.61–1.82	1.10	0.93–1.29
	Mixed fuel use	1.55	1.12–2.15	1.58	0.94–2.68	0.72	0.52–0.99	0.85	0.63–1.14	1.02	0.82

OR: odds ratio; 95% CI: 95% confidence interval; a Adjusted for age, BMI, marital status, highest level of education, smoking, alcohol consumption, physical activity and diabetes; b Self–reported diagnosis of angina pectoris.

Table S2. Angina treated.

Characteristic		Africa		Americas		Asia		Europe		Pooled sample	
		OR	(95% CI)	OR	(95% CI)	OR	(95% CI)	OR	(95% CI)	OR	(95% CI)
Household fuel used for cooking or heating 1	Gas	1		1		1		1		1	
	Electricity	1.07	0.71–1.60	0.34	0.05–2.22	0.93	0.46–1.92	0.37	0.26–0.53	0.64	0.50–0.83
	Kerosene	0.97	0.42–2.21	n/a	n/a	1.22	0.77–1.95	0.22	0.15–0.35	0.65	0.480.89
	Coal/charcoal	1.68	1.19–2.36	1.26	0.50–3.19	0.80	0.56–1.13	0.75	0.30–1.89	0.87	0.67–1.14
	Wood	1.83	1.39–2.40	0.89	0.60–1.31	0.95	0.73–1.23	0.86	0.53–1.39	1.02	0.84–1.23
	Agriculture/dung/shrubs/other	0.96	0.43–2.16	1.75	0.53–5.81	1.26	0.82–1.93	0.72	0.15–3.40	1.36	0.91–2.03
	Mixed solid/liquid/gas/electricity	1.19	0.72–1.97	1.95	0.93–4.10	0.88	0.60–1.29	0.87	0.62–1.23	1.21	0.95–1.53
	Mixed solid only	1.41	0.85–2.34	2.44	0.55–10.79	1.35	0.57–3.21	1.84	0.33–10.19	1.20	0.70–2.05
	Mixed liquid/gas/electricity only	0.96	0.39–2.33	2.48	1.03–6.01	0.77	0.51–1.16	0.53	0.33–0.85	0.91	0.60–1.38
Household fuel used for cooking or heating 2	Gas/electricity/kerosene	1		1		1		1		1	

Coal/charcoal/wood/ agriculture/crop/ shrub/grass	1.67	1.28–2.19	0.97	0.67–1.40	0.94	0.75–1.19	1.00	0.64–1.57	1.07	0.90–1.26
Mixed fuel use	1.17	0.80–1.71	2.22	1.30	0.92	0.67–1.28	0.85	0.64–1.12	1.14	0.92–1.40

Table S3. Rose questionnaire.

Characteristic		Africa		Americas		Asia		Europe		Pooled sample	
		OR	(95% CI)	OR	(95% CI)	OR	(95% CI)	OR	(95% CI)	OR	(95% CI)
Household fuel used for cooking or heating 1	Gas	1		1		1		1		1	
	Electricity	0.55	0.36–0.85	0.92	0.16–5.28	0.67	0.36–1.26	0.51	0.32–0.82	0.69	0.53–0.91
	Kerosene	0.83	0.45–1.54	n/a	n/a	1.55	1.11–2.16	0.24	0.16–0.37	1.04	0.81–1.34
	Coal/charcoal	0.75	0.53–1.06	1.31	0.68–2.53	0.54	0.41–0.71	1.68	0.72–3.93	0.81	0.65–1.00
	Wood	1.40	1.08–1.80	0.90	0.61–1.33	0.88	0.70–1.11	1.49	0.91–2.46	1.23	1.03–1.47
	Agriculture/dung/shrubs/other	2.26	1.27–4.00	1.33	0.58–3.05	1.33	1.01–1.76	1.69	0.60–4.77	1.90	1.47–2.47
	Mixed solid/liquid/gas/electricity	1.64	1.17–2.30	0.77	0.40–1.49	1.20	0.83–1.75	1.09	0.78–1.52	1.44	1.16–1.79
	Mixed solid only	2.02	1.46–2.80	1.36	0.59–3.16	1.24	0.61–2.51	0.18	0.04–0.83	1.86	1.30–2.66
	Mixed liquid/gas/electricity only	1.41	0.90–2.20	2.64	1.37–5.10	0.83	0.51–1.35	0.63	0.42–0.93	0.90	0.67–1.21
Household fuel used for	Gas/electricity/kerosene	1		1		1		1		1	

cooking or heating 2											
	Coal/charcoal/wood/agriculture/crop/shrub/ grass	1.75	1.37–2.23	0.98	0.69–1.39	0.85	0.71–1.02	1.78	1.14–2.77	1.23	1.07–1.41
	Mixed fuel use	2.36	1.81–3.07	1.53	0.90–2.60	1.13	0.82–1.56	1.01	0.78–1.31	1.30	1.10–1.53
